# An Uncommon Coronary Anomaly: A Single Right Coronary Artery With a Conus Branch Supplying the Left Anterior Descending Artery

**DOI:** 10.7759/cureus.69747

**Published:** 2024-09-19

**Authors:** Ahmet Kivrak, Mert Doğan, Ahmet Hakan Ates, Ugur Canpolat, Ahmet Aydin, Kudret Aytemir

**Affiliations:** 1 Cardiology, Faculty of Medicine, Hacettepe University, Ankara, TUR; 2 Cardiovascular System Surgery, Faculty of Medicine, Hacettepe University, Ankara, TUR

**Keywords:** conus artery, coronary vessel anomaly, non-st elevated myocardial infarction, single coronary artery, single right coronary artery

## Abstract

Coronary artery anomalies (CAAs) are usually asymptomatic and have a good prognosis. Single coronary artery anomaly (SCA) is a very rare CAA. Its true incidence is unknown. They are usually detected incidentally during coronary imaging and autopsy. Although most are asymptomatic, they can rarely cause clinical conditions such as sudden cardiac death and acute coronary syndrome (ACS). In this case report, a 44-year-old male patient was admitted to the emergency department with chest pain and coronary angiography revealed the absence of the left main coronary artery and a single right coronary artery (RCA). The patient's left system was fed by the posterior left ventricular artery (PLVA) and the conus artery. There was a serious lesion in the proximal PLVA and in the region close to the conus branch of the RCA. The patient underwent coronary artery bypass grafting and was discharged with recovery. In this case, we identified a single RCA in the patient presenting with ACS, and interestingly, the conus branch was supplying the proximal part of the left descending artery branch.

## Introduction

Coronary artery anomalies (CAAs) are generally asymptomatic and have a good prognosis [1]. They are usually detected incidentally during coronary angiography, autopsy, or cardiac computed tomography (CCT). The most common CAAs involve the left anterior descending coronary artery (LAD) and the circumflex artery (CX), which arise from separate ostia in the left sinus of Valsalva. An isolated single coronary artery anomaly (SCA) is very rare [2]. There is no clear algorithm yet for the management of SCA patients. Individualized treatment approaches are at the forefront according to the patient's complaint, clinical presentation, and coronary artery anatomy. These patients may present with sudden cardiac death or they may be asymptomatic and diagnosed incidentally. In addition, CAAs are one of the causes of sudden cardiac death in young athletes. In this report, we describe patient management and treatment in a case that presented with acute coronary syndrome (ACS) and was diagnosed as SCA.

## Case presentation

**Figure 1:**
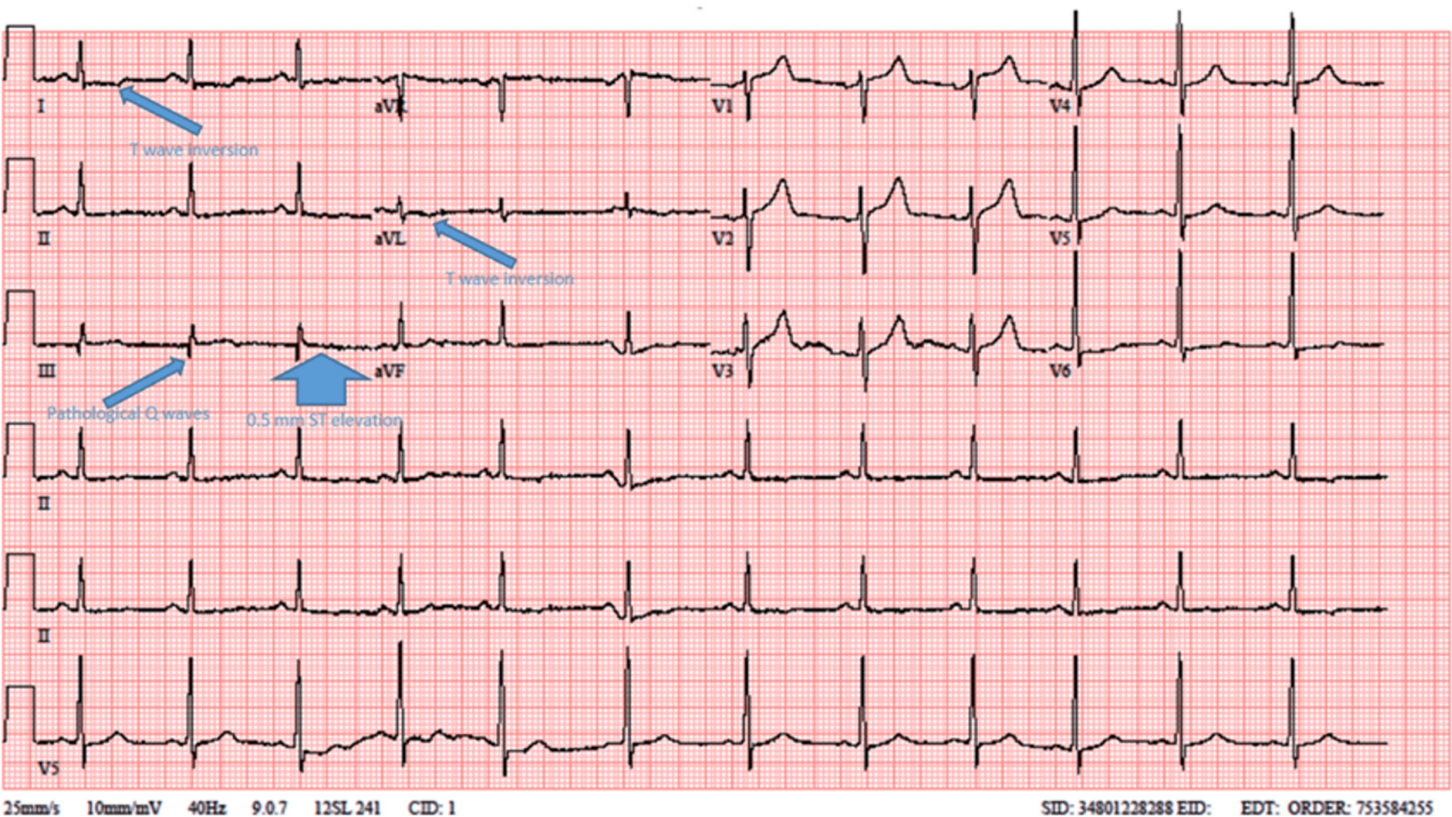
ECG at admission to the emergency room showing normal sinus rhythm, T-wave inversion in leads DI and aVL, and 0.5 mm ST elevation with pathological Q-waves in lead D3. ECG: electrocardiography

A 44-year-old male patient with a history of asthma presented to the emergency room with chest pain. The initial evaluation showed no abnormal findings during the physical examination. The patient’s 12-lead electrocardiography (ECG) revealed a heart rate of 73 beats per minute with a normal sinus rhythm, T-wave inversion in leads DI and aVL, and 0.5 mm ST elevation with pathological Q-waves in lead D3 (Figure 1). Echocardiography demonstrated an ejection fraction of 50%, with mild hypokinesia of the posterior and inferior walls.

**Figure 2:**
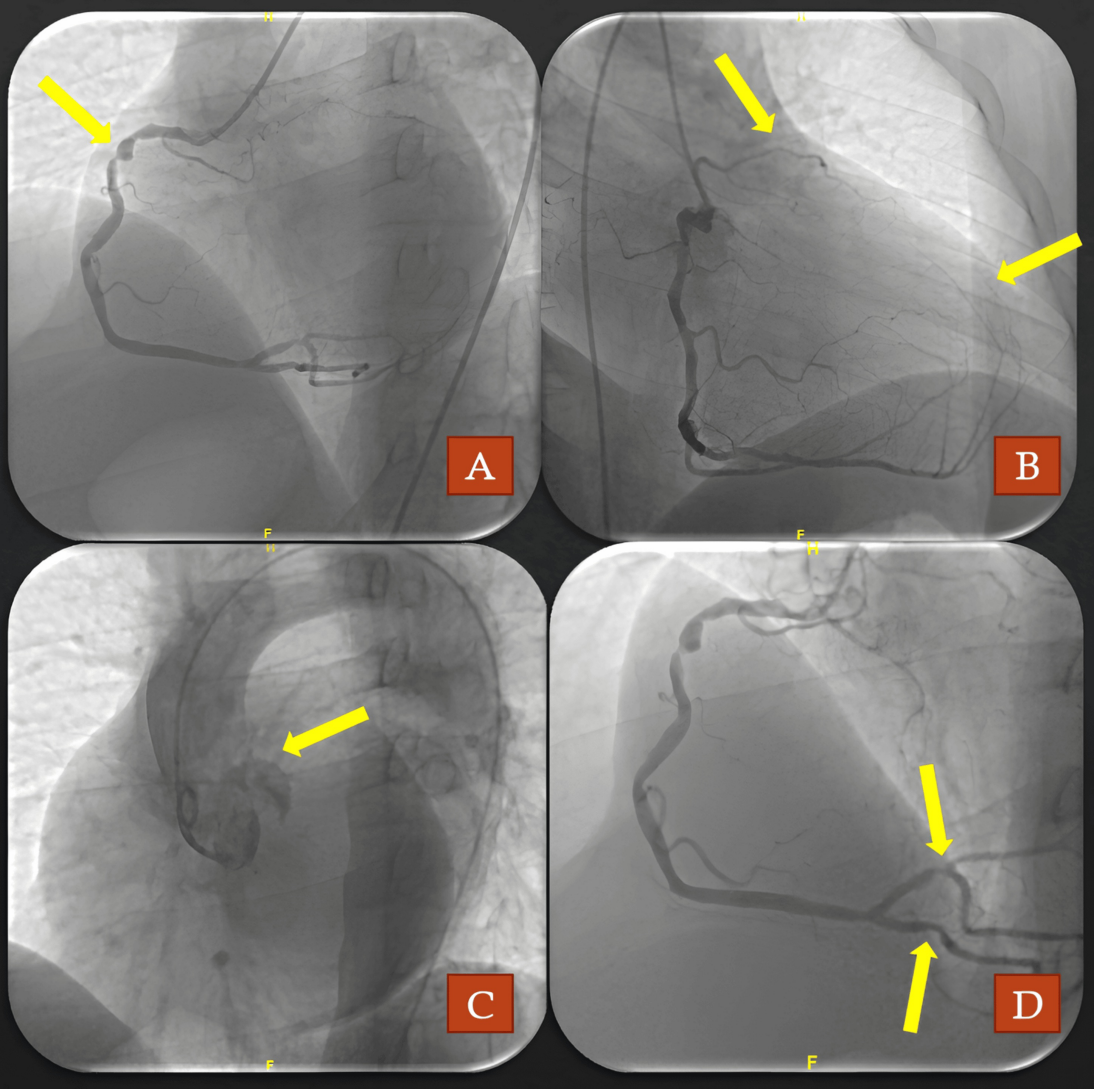
Coronary angiography images from different projections. (A) Left anterior oblique view (arrow); severe stenosis in the proximal RCA. (B) Right anterior oblique view (arrows); territory supplied by the LAD with branches originating from the RCA (upper arrow); RCA conus branch extends to the LAD region. (C) Aortography revealed absence of left coronary system arteries. (D) Severe stenoses in PDA and PLVA. LAD: left anterior descending coronary artery; RCA: right coronary artery; PLVA: posterior left ventricular artery; PDA: posterior descending artery

The laboratory results demonstrated normal values for complete blood count, liver function, and kidney function tests. Troponin-I was elevated at 190.8 ng/L (normal range: 14-42.9 ng/L). The patient was promptly taken to the interventional cardiology catheter laboratory for primary coronary intervention. A coronary angiograph was planned via right femoral access using a six-French sheath. Attempts to cannulate the left main coronary artery (LMCA) using left Judkins, Amplatz AL 1, Amplatz AL 2, and Amplatz AR 2 catheters (Cordis®) were unsuccessful. The right coronary artery (RCA) was visualized using a right Judkins catheter (Cordis®), revealing a lesion with 70% stenosis and another with 90% stenosis in the proximal RCA. Additionally, a 70% lesion was observed in the mid-region of the posterior descending artery (PDA) and another 70% lesion proximal to the posterior left ventricular artery (PLVA), which extended to the apex. Aortography was performed to visualize the LMCA, but no contrast filling was observed (Figures 2A-2D). It was understood that the LMCA was not cannulated because it had no ostium.

**Figure 3:**
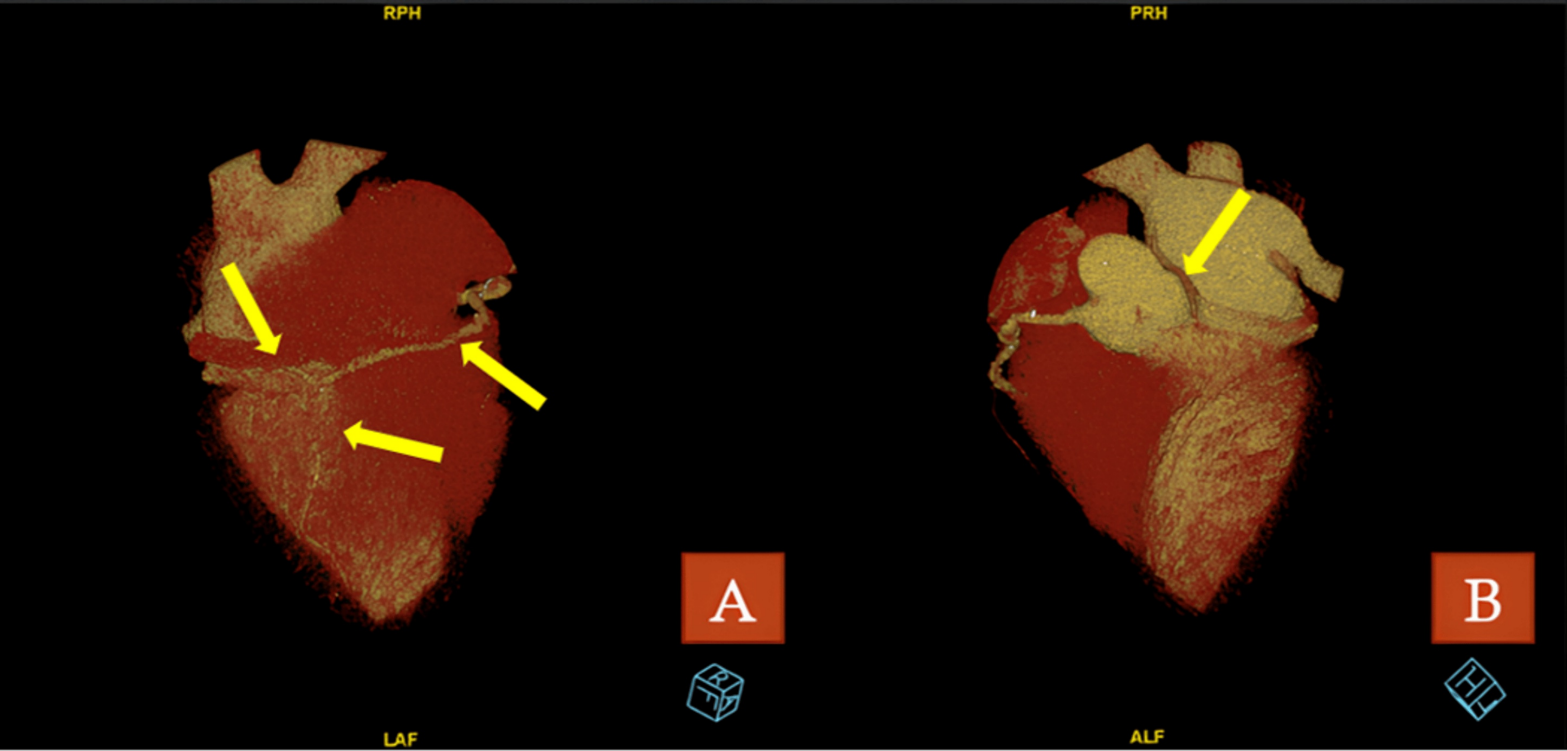
Three-dimensional reconstruction of coronary arteries in CCT angiography. (A) Severe stenoses in PDA and PLVA. (B) Absence of LAD and CX arteries, single RCA. CCT: cardiac computed tomography; LAD: left anterior descending coronary artery; CX: circumflex artery; RCA: right coronary artery; PLVA: posterior left ventricular artery; PDA: posterior descending artery

To better define the coronary anatomy, CCT was performed. It showed that the conus artery (1.5 mm in diameter) originated from the RCA and extended towards the left anterior descending territory at the mid-ventricular level (Figures 3A-3B). The patient was diagnosed with SCA. Since there was no major coronary artery supplying the left system and the PLVA along with the conus branch provided blood to the anterior wall, coronary artery bypass grafting (CABG) was deemed appropriate.

**Figure 4:**
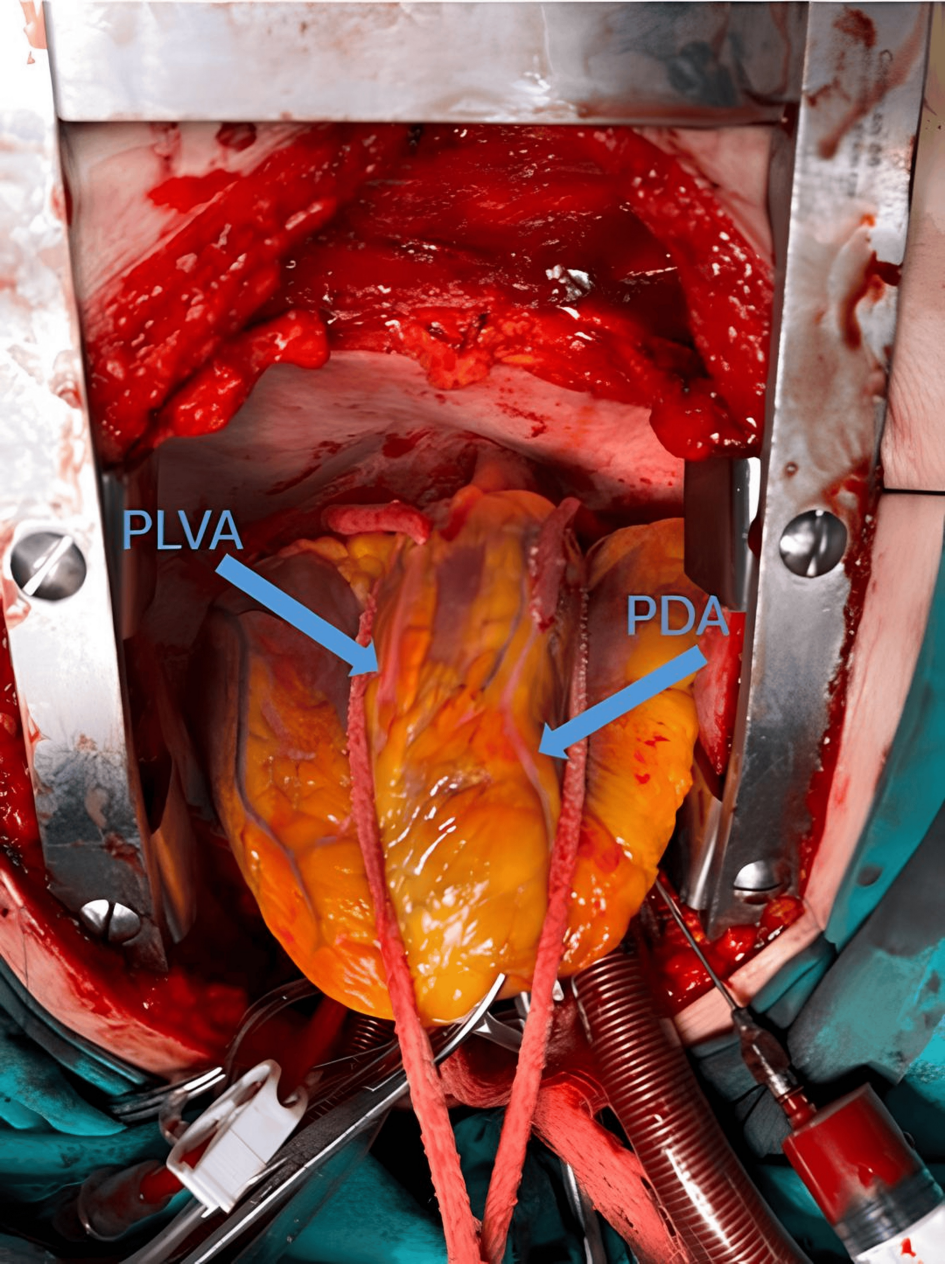
Intraoperative image showing RCA PLVA distal anastomosis, saphenous patchplasty of the PDA, and sequential saphenous-RCA-PDA distal anastomosis RCA: right coronary artery; PLVA: posterior left ventricular artery; PDA: posterior descending artery

During surgery, distal anastomosis of the RCA PLVA was performed followed by saphenous patchplasty on the PDA. The procedure was completed with sequential saphenous-RCA-PDA distal anastomosis (Figure 4). The patient was discharged after the appropriate medical treatment was arranged.

## Discussion

An SCA is a rare congenital anomaly characterized by the presence of only one coronary artery originating from the aortic sinus. Although its exact incidence is not well-established, a study reported its prevalence to be 0.044% [1]. Lipton's classification is used for the anatomical categorization of SCA based on coronary angiography findings [2]. In this classification system, R refers to the right sinus of Valsalva and L refers to the left sinus of Valsalva. Typically in type I, there is a solitary vessel that follows the course of a normal coronary artery (right or left), differentiating from a dominant right or left coronary artery, by continuing its course beyond the left atrioventricular groove or the crux, respectively. Type II describes an SCA originating from either the right or left sinus of Valsalva and passing through the base of the heart as a large transverse trunk to supply the opposite coronary artery. Type III involves an SCA originating from the right sinus of Valsalva, dividing into the LAD and CX arteries, which arise from separate trunks instead of a single trunk at the origin.

The subtypes are further categorized as follows:

A: SCA advancing in front of the right ventricle

B: SCA advancing between the aorta and pulmonary artery

P: SCA advancing behind the aorta and pulmonary artery

S: Septal type SCA

C: Combined type SCA

Our case was classified as RI type.

Although SCA is generally benign, some variants have been shown to increase the risk of sudden cardiac death [3]. While RI and LI are considered benign variants, the RII-III and LII-III types, which have a coronary artery branch coursing between the aorta and the pulmonary trunk, may predispose individuals to ischemia and arrhythmias. Type RII A passes anterior to the infundibulum and is considered a benign variant, without impeding reperfusion [4]. Rarely, patients with an RI anomaly may also develop a malignant course. In one reported case, it was shown to cause heart failure in a male patient who died at the age of 40 [5]. Additionally, an RI type coronary anomaly was detected during the autopsy of a six-year-old child who died after exercise [6]. Therefore, although RI and LI variants are generally considered benign, they have been associated with sudden cardiac death. In our case, the single RCA led to ischemia due to coronary artery disease, resulting in ACS in the patient.

Treatment management in SCA cases varies. The patient's symptoms, ECG, and echocardiography should be evaluated first. Revascularization should be considered in cases of myocardial ischemia, depending on the patient's symptoms and current clinical condition. The literature reports cases treated with pharmacological therapy, percutaneous coronary intervention (PCI), and CABG. There are documented cases where CABG has been successfully performed in SCA patients. There are reports in the literature showing that CABG has been successfully performed in SCA cases [7]. In our case, there was a significant lesion in the proximal RCA, and the conus branch appeared to originate near the lesion area. Given the conus artery's diameter of 1.5 mm, we anticipated difficulty in protecting the artery during PCI due to potential plaque shift. Therefore, we determined that CABG was the most appropriate treatment for the patient. The CABG procedure was successfully performed.

While the diagnosis of SCA can be conventionally made with coronary angiography, CCT and cardiac magnetic resonance imaging are important diagnostic tools for revealing detailed anatomy [8]. Coronary angiography remains the gold standard diagnostic method for coronary artery imaging. However, as demonstrated in our case, CCT is more useful in determining the anatomy in patients with ACS. It guides the revascularization strategy and can be performed more quickly than CMR. In our case, we found that the conus artery (1.5 mm in diameter) separated from the right coronary artery and extended towards the LAD irrigation area at the midventricular level.

A study on chronic total occlusions (CTO) showed that the conus branch is most frequently present in LAD CTO cases [9]. Although it could not be clearly demonstrated that the conus branch fed the LAD area, either due to long-term ischemia or congenitally, the anatomical examination during surgery revealed that while the PLVA branch mainly fed the left ventricle, the conus branch also extended to the LAD irrigation area. This likely indicates that in our case, where the left coronary artery system was congenitally absent, the conus branch supplied the left ventricle both congenitally and through subsequent collateral artery formation. In addition, a systematic review of the Vieussens' arterial ring, which is a ring-shaped anastomosis between the conus branch and the LAD, suggested anatomical classification [10]. According to the anatomical classification, the conus branch, which originates from the proximal RCA, extends to the proximal LAD region and is classified as type IA Vieussens' arterial ring. To our knowledge, this is the first case presenting with ACS, showing that the conus branch feeds the proximal LAD and the PLVA feeds the distal part of the LAD.

## Conclusions

SCA is a very rare CAA that is usually diagnosed incidentally. These patients may be asymptomatic or present with conditions such as sudden cardiac death. In cases of ACS developing on the basis of SCA, the revascularization strategy should be carefully selected. In fact, there is no treatment algorithm yet for SCAs diagnosed on the basis of ACS. In this article, we presented a case in which the conus artery originating from the proximal RCA fed the proximal LAD region. Due to the risk of plaque shift, we decided that CABG treatment was more appropriate than PCI and the patient underwent surgery. Clinically personalized treatment methods should be applied in cases of SCA. At the same time, this case is valuable as it is the first case showing that the conus artery originating from a single RCA presenting on the basis of ACS extended to the proximal LAD.
